# Analyzing the Ratio Between Prenatal Consultations and Live Births in Brazil: Spatial and Professional Patterns, 2008–2023

**DOI:** 10.1002/puh2.70387

**Published:** 2026-10-07

**Authors:** Márcio Cristiano de Melo, Jucilene Casati Lodi, Luiz Eduardo Coelho Emerenciano, Nayara Amelia Guirau Veronez, Tassia Fraga Bastos, Naila Albertina de Oliveira

**Affiliations:** ^1^ São Leopoldo Mandic School of Medicine Araras São Paulo Brazil; ^2^ São Leopoldo Mandic School of Medicine Limeira São Paulo Brazil; ^3^ São Leopoldo Mandic School of Medicine Campinas São Paulo Brazil

**Keywords:** live birth, prenatal care, primary health care, spatial analysis

## Abstract

This study analyzed the ratio between antenatal care visits and live births (LB) in Brazil as an indicator of service utilization intensity. This nationwide ecological longitudinal study combined descriptive temporal and spatial analyses of outpatient antenatal consultations recorded in SIA/SUS and LB reported in SINASC across Brazilian federative units and macroregions from 2008 to 2023. The ANC/LB ratio was interpreted as an indirect measure of the intensity of consultation utilization and recording relative to LB, rather than as an individual‐level measure of antenatal care coverage or adequacy. An increase in the ANC/LB ratio was observed until 2014, followed by fluctuations and lower values from 2020 onward, with partial increases in some regions in subsequent years. Spatial analysis showed marked geographic variation, with higher ratios more frequently observed in the South and Southeast and lower values in several states in the North and Northeast. In 2023, nurses accounted for the largest proportion of antenatal consultations nationwide, particularly in the North and Northeast, whereas obstetrician–gynecologists had greater relative participation in the Southeast. Limitations include potential variation in the quality and completeness of SIA/SUS records, which may affect temporal and geographic comparability. These findings provide a nationwide perspective on the recorded intensity and professional composition of antenatal care and may support monitoring and health‐service planning.

## Graphical abstract

Ecological analysis of the antenatal care consultation‐to‐live birth (ANC/LB) ratio across Brazil's 27 federal units (2008–2023) identified an overall increase before COVID‐19, followed by a pandemic‐related decline and heterogeneous recovery from 2021 onward, highlighting persistent regional disparities and the need to strengthen equitable antenatal care.

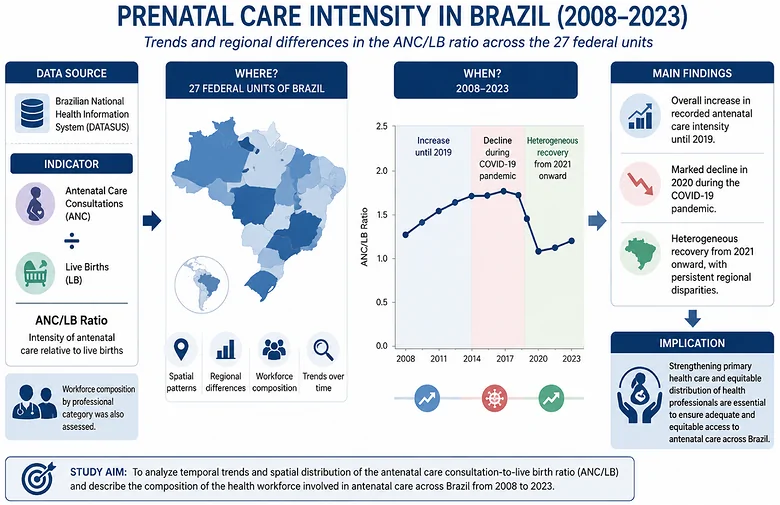

## 1 Introduction

The quality of care delivered during pregnancy, childbirth, and the postpartum period is a central pillar of maternal health and a key determinant of perinatal outcomes. In Brazil, following the establishment of the Unified Health System (Sistema Único de Saúde—SUS) and the expansion of the Family Health Strategy (Estratégia Saúde da Família—ESF), national policies have sought to strengthen maternal and child health through structured care models. Notable initiatives include the Program for the Humanization of Prenatal and Birth Care (PHPN 2000) [1] and the Rede Cegonha (Stork Network), launched in 2011, which advanced an integrated framework for antenatal, intrapartum, and postpartum services. Core components include early initiation of antenatal care (before 12 weeks of gestation), completion of a minimum number of visits, referral linkage to maternity services, and the right to a companion during childbirth. These strategies align with World Health Organization recommendations advocating continuous, comprehensive, and person‐centered care that encompasses prevention, timely access, diagnosis, treatment, information, and social support to improve maternal and perinatal outcomes [2].

Brazil maintains robust maternal and child health surveillance and evaluation systems, notably the Live Birth Information System (Sistema de Informações sobre Nascidos Vivos—SINASC) and the Outpatient Information System of the Unified Health System (Sistema de Informações Ambulatoriais do SUS—SIA/SUS). These nationwide administrative databases enable large‐scale monitoring of antenatal care delivery and utilization, supporting epidemiological assessment and informing evidence‐based public policy across the country [3, 4, 5].

A particularly relevant metric in this context is the ratio of antenatal care visits to live births (LB), interpreted as an indirect indicator of service utilization rather than individual‐level coverage. This indicator provides a synthetic and comparable measure of antenatal service delivery over time and across geographic regions. Its examination allows assessment not only of service coverage and utilization, but also of potential disparities in access, the timeliness of care, and the continuity of antenatal follow‐up across different areas of the country [6, 7].

The methodological approach combining descriptive temporal analysis from 2008 to 2023 with detailed spatial assessment enhances the capacity to identify temporal variation and regional differences in antenatal care delivery. This integrated analytical framework enables the detection of temporal shifts associated with structural determinants, including economic cycles, health system reorganization, and large‐scale systemic shocks, such as the COVID‐19 pandemic [3, 6, 7].

The application of these analytical methods is critical to informing the formulation and implementation of maternal and child health policies. By providing a comprehensive understanding of temporal and regional variation in antenatal care delivery, this approach supports the development of more targeted and context‐sensitive interventions. Ultimately, strengthening the quality and equity of antenatal care has the potential to improve maternal and fetal outcomes nationwide, reduce health disparities, and advance progress in key maternal and child health indicators across Brazil [6, 7].

Additionally, the professional composition of antenatal consultations—physicians, nurses, and other health professionals—represents a critical component of the care process. Both scientific literature and national guidelines emphasize the value of multiprofessional models, with complementary roles in screening, health education, clinical management, and risk stratification. Mapping who delivers antenatal care and how this distribution varies across regions and over time provides insight into health system capacity, models of care organization, and potential barriers to access. Such analysis can inform workforce planning, quality improvement strategies, and the equitable allocation of human resources in maternal health services [3, 6, 7].

The aim of this study was to analyze the ratio between the number of antenatal care visits and LB in Brazil from 2008 to 2023, examining spatial variation across regions and according to the professional category responsible for care delivery, using data from the SINASC and the Outpatient Information System of the Unified Health System (SIA/SUS).

## 2 Methods

We conducted a nationwide ecological longitudinal time‐series study integrating spatial analyses using secondary administrative data aggregated at the level of Brazil's 27 federative units and by macroregion, from 2008 to 2023. The primary unit of analysis was the federative unit. We examined the ratio of antenatal care visits recorded in the SUS to LB, assessing temporal and geographic variation in this indicator. Additionally, we analyzed the distribution of professional categories delivering antenatal care to characterize workforce composition and its variation over time and across regions.

Data were obtained from the Department of Informatics of the Unified Health System (DATASUS) and from two publicly available national information systems. The SINASC, accessed through the vital statistics section, provided annual counts of LB according to the mother's place of residence. From the Outpatient Information System (SIA/SUS), specifically the consolidated Outpatient Production Bulletin (BPA‐C), we extracted records of ambulatory procedures related to the pregnancy‐puerperal cycle, including antenatal care visits (low‐risk and high‐risk pregnancies) and related services, such as partner consultation and high‐risk postpartum visits, according to the procedure codes in force during the study period. No formal imputation procedures were applied. Missing or inconsistent records were assessed through internal consistency checks and interpreted cautiously.

To ensure epidemiological consistency, the antenatal visits‐to‐live births ratio (ANC/LB) was interpreted as a measure of ambulatory care intensity related to pregnancy and the puerperium rather than as a direct estimate of antenatal care coverage. The analysis further incorporated the distribution of antenatal consultations according to the Brazilian Classification of Occupations (CBO) for 2023, the last year of the study period, enabling a cross‐sectional characterization of the professional categories responsible for antenatal care delivery. This analysis was intended to provide contextual information on the workforce involved in antenatal care rather than to assess temporal changes in workforce composition.

The primary study variable was the ratio between the number of antenatal care visits and the number of LB, calculated annually for each federative unit using the following formula:

$$\begin{array}{ccc} & & \mathrm{ANC}/\mathrm{LB}\;\mathrm{ratio}\\ & & =\frac{\mathrm{Number}\;\mathrm{of}\;\mathrm{recorded}\;\mathrm{antenatal}\;\mathrm{care}\;\mathrm{visits}}{\mathrm{Number}\;\mathrm{of}\;\mathrm{live}\;\mathrm{births}\;\mathrm{in}\;\mathrm{the}\;\mathrm{same}\;\mathrm{period}\;\mathrm{and}\;\mathrm{geographic}\;\mathrm{unit}} \end{array}$$

The numerator included outpatient procedures coded as antenatal care visits for low‐ and high‐risk pregnancies. Procedures not directly attributable to antenatal follow‐up—such as partner consultations and postpartum visits—were excluded from the numerator of the primary indicator.

The ANC/LB ratio was defined as the annual ratio of recorded outpatient antenatal visits to LB within the same geographic unit and period. This measure was interpreted as an indirect indicator of the intensity of antenatal consultation utilization and registration relative to LB. It is suitable for spatial and temporal comparisons; however, it does not correspond to the average number of visits per pregnancy at the individual level.

The analysis was conducted in two stages. First, we conducted a descriptive temporal analysis of the ANC/LB ratio from 2008 to 2023, examining changes in the observed values by macroregion. Second, we conducted a descriptive spatial analysis, constructing choropleth maps for 2008, 2011, 2014, 2017, 2020, and 2023 to depict geographic variation in the ratio of antenatal care visits to LB across Brazil's federative units.

Given the potential for operational variability within administrative information systems, particularly in SIA/SUS (consolidated BPA records), we conducted consistency checks of annual totals by federative unit and inspected extreme values and abrupt series breaks through internal comparisons across years and between states. Observed fluctuations were interpreted in light of possible changes in coding practices, recording and billing routines, and differential underreporting, especially during the COVID‐19 pandemic, which may have affected temporal comparability across specific states.

Thematic maps were produced using R (R Foundation for Statistical Computing, Vienna, Austria), version 4.3.3, ensuring analytical reproducibility and standardization of cartographic procedures. Official territorial shapefiles for Brazilian federative units were imported using the geobr package, which provides vector files aligned with administrative boundaries defined by the Brazilian Institute of Geography and Statistics (IBGE). Spatial data processing and integration with the analytical dataset were performed using the sf package. Data organization and transformation were conducted with dplyr and tidyr. To facilitate comparisons across time, all choropleth maps were classified using fixed class intervals based on the global distribution of the ANC/LB ratio throughout the study period. The following intervals were applied consistently to all maps: 0.0–5.0, 5.1–10.0, 10.1–15.0, 15.1–20.0, 20.1–25.0, 25.1–30.0, and >30.0. Cartographic visualization was generated with the tmap package, employing a monochromatic grayscale scale with uniform state borders. Outputs were exported in high‐resolution PNG format (300 dpi) and as multipage PDF files for scientific dissemination. The publisher remains neutral regarding jurisdictional claims in published maps.

Data were tabulated and analyzed descriptively, with frequencies and proportions reported for categorical variables. Temporal variation was assessed descriptively by examining annual ANC/LB ratio values within each macroregion and federative unit and describing increases, decreases, and fluctuations over the study period. Spatial analysis compared geographic patterns across regions and selected years, highlighting areas with higher or lower antenatal care delivery intensity. All statistical analyses and graphical procedures were conducted in R, ensuring reproducibility of data tabulation and visualization. This study was reported in accordance with the Strengthening the Reporting of Observational Studies in Epidemiology (STROBE) guidelines.

## 3 Results

Table 1 presents the evolution of the number of antenatal care visits (ANC) and LB across all Brazilian federative units from 2008 to 2023. Antenatal consultations increased overall until 2014, although the magnitude of the increase varied substantially across states. States, such as São Paulo, Minas Gerais, Bahia, and Pernambuco, consistently recorded the highest absolute numbers of antenatal visits throughout the study period.

**TABLE 1:** Antenatal care consultation‐to‐live birth (ANC/LB) ratio by Brazilian state in the selected study years, Brazil, 2008–2023.

Brazilian federative unit (state)	ANC/LB ratio					
	2008	2011	2014	2017	2020	2023
Rondônia	2.79	5.50	6.13	3.69	1.30	1.48
Acre	3.89	32.75	4.95	4.71	2.07	2.12
Amazonas	4.40	4.34	9.18	3.92	0.22	0.11
Roraima	4.32	7.69	4.25	11.43	0.05	0.08
Pará	8.48	6.55	10.99	6.56	1.30	0.16
Amapá	5.13	5.41	7.18	3.17	0.02	0.00
Tocantins	14.15	5.74	10.20	4.77	0.94	0.44
Maranhão	14.08	12.37	11.78	8.39	2.88	1.17
Piauí	3.15	6.92	8.04	5.58	0.10	0.06
Ceará	4.19	5.71	8.89	12.97	0.51	0.25
Rio Grande do Norte	4.90	12.66	4.50	5.31	3.25	0.17
Paraíba	8.41	8.08	9.35	3.75	0.31	0.30
Pernambuco	4.73	9.21	10.02	4.78	0.50	0.41
Alagoas	5.03	4.93	6.51	2.81	0.07	0.05
Sergipe	17.10	4.05	9.28	4.39	1.12	0.51
Bahia	7.63	11.44	11.02	7.33	0.41	0.15
Minas Gerais	5.49	5.70	12.26	6.88	2.36	1.33
Espírito Santo	3.81	6.29	12.60	4.85	3.30	0.67
Rio de Janeiro	4.59	3.44	4.54	5.15	2.12	5.32
São Paulo	6.49	4.84	7.79	6.55	3.54	3.15
Paraná	3.84	6.36	5.55	9.60	3.09	3.52
Santa Catarina	4.67	10.74	12.38	4.83	2.63	3.01
Rio Grande do Sul	3.16	4.78	12.40	3.33	1.56	1.30
Mato Grosso do Sul	4.29	4.00	4.76	4.56	2.02	1.55
Mato Grosso	11.27	11.30	20.36	7.76	2.45	1.38
Goiás	3.72	5.05	16.39	4.41	1.11	1.58
Distrito Federal	6.79	4.53	4.55	5.32	0.59	0.45
Brazil	6.18	6.73	9.38	6.32	1.98	1.76

*Note:* The corresponding absolute numbers of antenatal care consultations and live births used to calculate the ANC/LB ratio are presented in Table S1.

*Source:* Authors’ analysis based on data from SIA/SUS and SINASC, DATASUS, Brazil.

In contrast, federative units in the North region, such as Amapá and Roraima, consistently recorded the lowest absolute numbers of antenatal visits and LB. From 2020 onward, substantial reductions in the recorded ANC/LB ratio were observed across several states, with decreases ranging from approximately 50% to over 70% in some federative units (Table 1).

Figure 1 shows the spatial distribution of the ANC/LB ratio across Brazilian states in 2008, 2011, 2014, 2017, 2020, and 2023. In 2008, higher ratios were observed in the Northeast and Central‐West regions, with Maranhão and Mato Grosso among the states with higher values. By 2011, increases in the ratio were observed across several states, with Acre recording the highest value during the study period.

**FIGURE 1:**
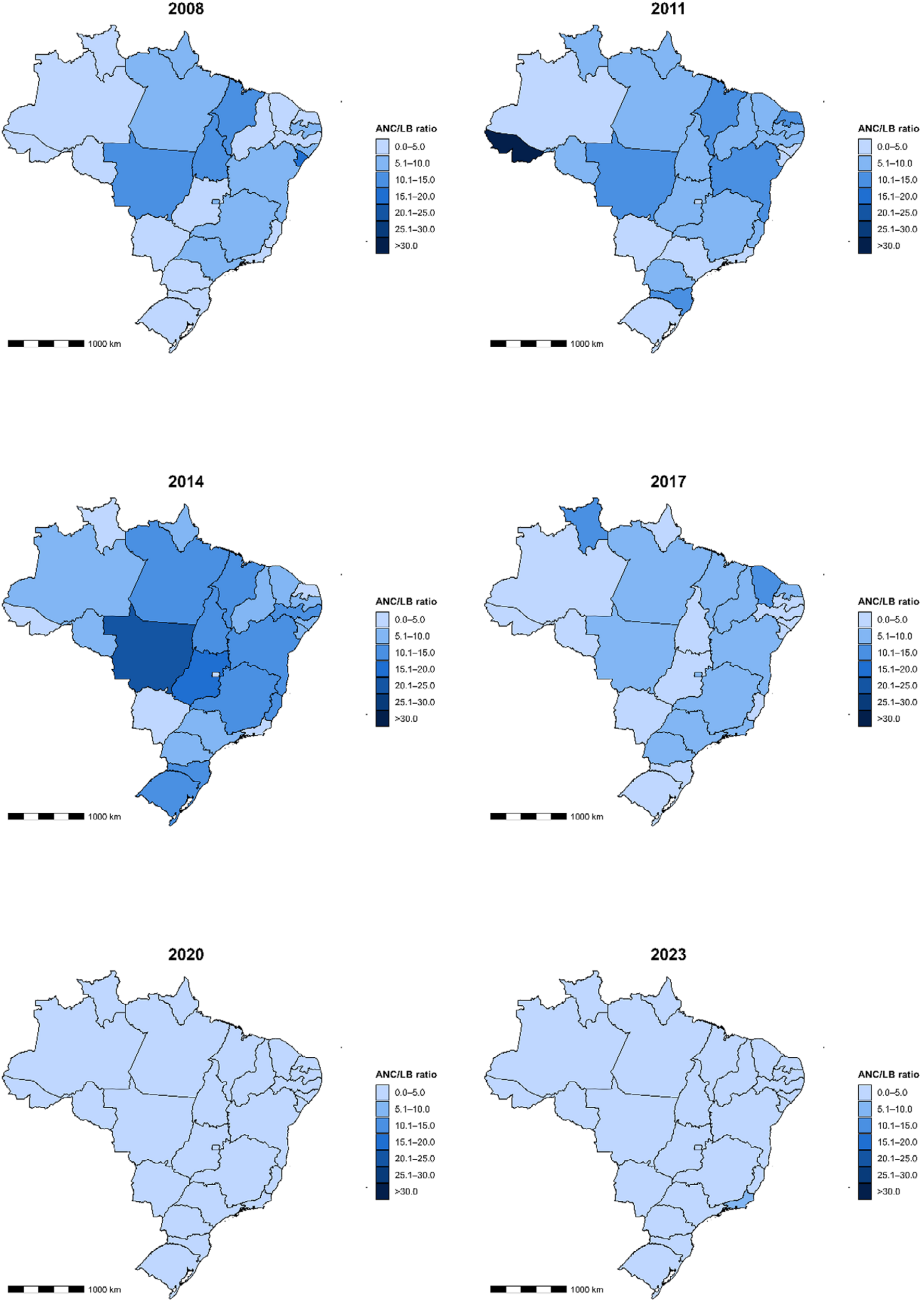
Spatial distribution of the antenatal care consultation‐to‐live birth (ANC/LB) ratio across Brazilian states, 2008–2023. Fixed class intervals were applied to all maps to allow direct comparison between study years.

In 2014, the spatial distribution of the ANC/LB ratio was more homogeneous, with higher values observed in several Southeastern and Southern states, including Minas Gerais, São Paulo, and Santa Catarina. By 2017, higher ratios remained evident in several states in the South and Southeast, whereas lower values were observed in several states in the North and Northeast (Figure 1).

In 2020 and 2023, lower ANC/LB ratios were observed across most federative units. Higher ratios were more frequently observed during the earlier years of the series, whereas from 2020 onward, lower ANC/LB ratio categories were observed in most states (Figure 1).

Table 2 presents the distribution of professional categories responsible for antenatal consultations in Brazil in 2023, stratified by macroregion. Nurses accounted for the largest share of antenatal visits nationwide, representing 46.3% of all consultations, with higher proportions observed in the Northeast (68.0%) and North (61.3%) regions. General practitioners and family physicians accounted for 27.5% of consultations nationwide, with higher proportions in the Central‐West (39.8%) and South (34.7%) regions.

**TABLE 2:** Distribution of antenatal care consultations by professional category and Brazilian macroregion, 2023.

	North region		Northeast region		Southeast region		South region		Central‐West region		Brazil	
	*N*	%	*N*	%	*N*	%	*N*	%	*N*	%	*N*	%
Nurses	17,325,343	61.32	54,659,261	68.02	28,705,824	30.73	9,108,476	29.04	8,843,760	38.62	118,642,664	46.29
GPs and family physicians	7,781,845	27.54	20,913,354	26.02	21,807,088	23.35	10,874,320	34.67	9,122,444	39.83	70,499,051	27.51
Maternal–child physicians and OB–GYNs	3,125,197	11.06	4,738,021	5.9	42,842,069	45.87	11,371,630	36.26	4,929,463	21.52	67,006,380	26.15
Nursing techs and assistants	10,547	0.04	12,074	0.02	25,015	0.03	816	0	2954	0.01	51,406	0.02
Physicians—clinical specialties	3427	0.01	8137	0.01	3965	0	1267	0	1623	0.01	18,419	0.01
Physicians—diagnostic and therapy	3634	0.01	6778	0.01	1070	0	781	0	811	0	13,074	0.01
Dentists and oral health assistants	3292	0.01	2676	0	3702	0	479	0	489	0	10,638	0
Surgeons	467	0	4643	0.01	4938	0.01	444	0	85	0	10,577	0
Primary care support staff	631	0	4976	0.01	3758	0	698	0	39	0	10,102	0
Community health workers (CHWs)	474	0	4956	0.01	2986	0	687	0	39	0	9142	0
Clinical and social support staff	527	0	4265	0.01	204	0	28	0	0	0	5024	0
Rehabilitation professionals	67	0	1828	0	110	0	8	0	0	0	2013	0
Psychiatrists and neurologists	2	0	146	0	582	0	528	0	358	0	1616	0
Public health physicians	28	0	35	0	124	0	663	0	1	0	851	0
Other specialized technicians	0	0	0	0	0	0	1	0	18	0	19	0
Total	28,255,481	100	80,361,150	100	93,401,435	100	31,360,826	100	22,902,084	100	256,280,976	100

*Note:* Nurses; general practitioners and family physicians; maternal–child physicians and obstetrician–gynecologists; nursing technicians, assistants, and attendants; physicians in clinical specialties; physicians in diagnostics and therapeutic services; dentists and oral health assistants; surgeons; primary care support professionals; community health workers; clinical and social support professionals; rehabilitation professionals; psychiatrists and neurologists; public and collective health physicians; and other specialized technical professionals. These categories reflect the diversity of the health workforce, encompassing roles across primary care, comprehensive maternal health services, clinical and surgical specialties, diagnostic services, and technical support functions, highlighting the multidisciplinary structure required for effective health system performance.

*Source:* Authors’ analysis based on data from SIA/SUS, DATASUS, Brazil.

Maternal–child health physicians and obstetrician–gynecologists accounted for 26.1% of antenatal consultations nationwide, with the highest proportion observed in the Southeast (45.9%). Other professional categories—including nursing technicians, nursing assistants, clinical and social support staff, and community health workers (CHWs)—each represented less than 1% of total consultations (Table 2).

## 4 Discussion

This nationwide study identified substantial temporal and geographic variation in the recorded ANC/LB ratio across Brazilian federative units between 2008 and 2023. Higher values were observed during the earlier years of the study period, followed by lower values across most federative units from 2020 onward. The spatial distribution varied across regions and over time, whereas the 2023 professional profile showed that nurses accounted for the largest proportion of antenatal consultations nationwide, with marked regional differences in workforce composition.

The Previne Brasil program, implemented in 2019, introduced substantial changes to the financing and organization of primary health care (PHC) in Brazil, with potential implications for antenatal care indicators. By establishing performance‐based payments tied to specific metrics—including the proportion of pregnant women completing at least six antenatal visits—the program may have incentivized greater attention to gestational follow‐up within primary care teams. Such policy shifts are consistent with intensified active outreach, systematic appointment scheduling, and closer monitoring of antenatal care delivery. The increases observed in the intensity of recorded antenatal consultations in several regions may partly reflect these structural incentives, although regional and socioeconomic inequalities remain persistent challenges [8].

Table 1 demonstrates a marked increase in the number of antenatal consultations between 2008 and 2014, followed by subsequent fluctuations through 2023. The increase observed between 2008 and 2014 coincides with a period of expansion and strengthening of PHC in Brazil, including the implementation of national initiatives, such as Rede Cegonha and the Mais Médicos (More Doctors) programs. These policies were designed to expand access, improve continuity of care, and enhance the quality of maternal health services and have been associated in previous studies with increased proportions of women completing six or more antenatal visits and reductions in regional disparities [3, 7, 9, 10]. Although causality cannot be inferred from the present ecological design, the temporal pattern observed is consistent with health system strengthening efforts; however, this association should not be interpreted as evidence of a causal relationship.

After 2014, the ANC/LB ratio fluctuated, with lower values observed from 2020 onward. Evidence from national and international studies indicates that the emergency reorganization of health services during the COVID‐19 pandemic substantially disrupted routine antenatal care delivery. Temporary closures of health facilities, reallocation of resources, and pregnant women's reluctance to attend in‐person appointments were reported in several settings, leading to interruptions in gestational follow‐up and potential adverse implications for perinatal outcomes and the management of maternal conditions [9, 10, 11]. The decline in the recorded ANC/LB ratio is temporally consistent with the COVID‐19 pandemic.

The gradual recovery in antenatal consultations observed in subsequent years indicates a degree of resilience within Brazil's PHC system. However, this recovery was heterogeneous across regions—more rapid in the Southeast and Central‐West and slower in the North [6, 9, 10, 12]. These findings are consistent with longstanding patterns of regional inequality in Brazilian antenatal care provision, historically associated with the unequal distribution of financial resources, limited physical infrastructure, and disparities in the health workforce [13].

Increases in the number of antenatal visits alone do not necessarily indicate improvements in the quality or comprehensiveness of care. The postpartum follow‐up protocol of the Birth in Brazil II study incorporates assessments of maternal and paternal mental health, maternal and neonatal morbidity, use of postnatal services, and experiences of maternity care [14]. This broader assessment framework highlights dimensions of maternal and child health that are not captured by consultation counts alone.

The Rede Alyne, established by the federal government in 2024 to replace the former Rede Cegonha, represents a renewed national strategy to strengthen maternal and child health care. The policy emphasizes professional qualifications, quality improvement, and equity‐oriented approaches, with particular attention to socially and racially vulnerable populations [15]. By reinforcing the longitudinality of care—a core principle of PHC—it seeks to ensure continuous follow‐up of pregnant women through multidisciplinary health teams and CHWs, thereby promoting sustained engagement and improving the quality of antenatal services [16].

Despite progress, Brazil remains far from the targets set under the Sustainable Development Goals (SDGs), which aim to improve the quality and equity of maternal health care by 2030. Strengthening PHC through sustained financial investment and enhanced integration of nurses and CHWs could yield substantial gains. As the level of care responsible for ensuring continuity, coordination, and long‐term patient engagement, PHC constitutes a cornerstone of the health system and plays a critical role in improving maternal and child health indicators while reducing inequities [17].

The predominance of nurses in antenatal consultations observed in 2023, the final year of the study period, reflects the expansion of their clinical scope and the consolidation of PHC as the central axis of maternal and child health care in Brazil. The growing participation of nurses—particularly in the North and Northeast regions—is aligned with the advancement of policies promoting Advanced Nursing Practice, which strengthen professional autonomy and expand the role of nurses in antenatal follow‐up, breastfeeding promotion, and child development monitoring [18, 19, 20, 21].

This shift aligns with the international trend toward integrating Advanced Practice Nurses (APNs) into complex clinical roles, enhancing the continuity, comprehensiveness, and problem‐solving capacity of care—particularly in settings with physician shortages [19, 20]. In Brazil, the performance of nursing professionals in antenatal care has also been reinforced by pay‐for‐performance financing strategies, such as the former National Program for Improving Primary Care Access and Quality (PMAQ‐AB) and the Previne Brasil program, which prioritize antenatal follow‐up indicators and encourage territorially integrated clinical practices [22].

The cross‐sectional profile presented in Table 2 underscores that the consolidation of antenatal care in Brazil depends not only on expanding PHC coverage but also on strengthening the qualification, recognition, and equitable distribution of the nursing workforce across regions. These elements are essential to ensuring continuity of care and reducing maternal and perinatal inequalities [23].

The substantial participation of general practitioners and family physicians, followed by obstetrician–gynecologists, indicates the coexistence of distinct care models across Brazilian regions. In the South and Southeast, maternal health services are more frequently supported by structured referral networks with a stronger presence of medical specialists, whereas the North and Northeast rely more heavily on community‐based and multiprofessional primary care models centered on comprehensive care [13, 23, 24]. This heterogeneous regional configuration has implications for the quality and equity of access, as recent workforce distribution studies indicate a concentration of highly specialized professionals in urban centers and persistent shortages in less densely populated regions [23].

This study has limitations. It relied on secondary data from official health information systems, which are subject to underreporting, regional inconsistencies, and variation in data quality and completeness. In particular, the numerator of the ANC/LB ratio was derived from consolidated BPA records from SIA/SUS and may have been affected by changes in reporting and billing practices, procedure coding, and heterogeneous underreporting across federative units over time. Consequently, some observed variations, including low values in specific states and periods, may reflect differences in data recording and completeness rather than changes in antenatal care utilization alone. Aggregation at the federative‐unit level may also obscure relevant intraregional disparities, particularly in small or geographically remote municipalities.

Moreover, the use of aggregated temporal and spatial indicators precludes causal inference about the relationships between observed variations and structural determinants such as workforce availability, health financing, or local policy implementation. Despite these limitations, the study provides a nationwide and comparable overview of the recorded intensity and professional composition of antenatal care in Brazil and may inform future research using more granular data and analytical approaches.

The findings show increases in the recorded ANC/LB ratio between 2008 and the mid‐2010s, followed by fluctuations and lower values during the COVID‐19 pandemic.

The temporal and spatial analysis of the ANC/LB ratio revealed persistent regional inequality, with more favorable patterns in the South and Southeast and consistently lower values in the North and Northeast, where greater socioeconomic vulnerability and lower service density may have amplified the effects of the health crisis. Recovery from 2021 onward was heterogeneous, indicating that institutional resilience, the adaptive capacity of PHC, and broader public health responses may have contributed to the gradual restoration of antenatal care intensity.

The professional composition of antenatal care confirms the central role of nurses and general practitioners, highlighting their expanded potential in strategies to restore antenatal care following the disruption caused by the COVID‐19 pandemic. These include proactive outreach to pregnant women, complementary telehealth consultations when clinically appropriate, reorganization of schedules to prioritize early booking and low‐risk follow‐up, and effective integration with referral networks for high‐risk pregnancies.

This study identified substantial temporal and regional variation in the intensity of recorded antenatal care relative to LB across Brazil from 2008 to 2023, as well as marked geographic differences in the professional composition of antenatal care. These findings provide a nationwide perspective on antenatal care delivery and underscore the need to strengthen PHC, promote equitable distribution of the workforce, and improve the quality and completeness of health information systems.

## Author Contributions

**Márcio Cristiano de Melo**: conceptualization, investigation, writing – original draft, validation, visualization, writing – review and editing, formal analysis, project administration, supervision, resources, funding acquisition, methodology, data curation, software. **Jucilene Casati Lodi:** conceptualization, visualization, writing – review and editing, data curation. **Luiz Eduardo Coelho Emerenciano**: visualization, writing – review and editing, data curation. **Nayara Amelia Guirau Veronez**: visualization, writing – review and editing, data curation. **Tassia Fraga Bastos**: visualization, writing – review and editing, data curation. **Naila Albertina de Oliveira**: conceptualization, investigation, validation, visualization, formal analysis, data curation, supervision, writing – review and editing, methodology.

## Funding

The authors have nothing to report.

## Ethics Statement

This study used exclusively publicly available, aggregated secondary data and did not involve access to individually identifiable information. Therefore, ethics committee approval and informed consent were not required, in accordance with applicable Brazilian regulations.

## Conflicts of Interest

The authors declare no conflicts of interest.

## Supporting information




**Supporting File 1:** Puh270387‐sup‐0001‐S1.docx.

## Data Availability

The data that support the findings of this study are openly available in DATASUS at https://datasus.saude.gov.br/ and SINASC databases.
