# Supplementary material for: Analyzing the Ratio Between Prenatal Consultations and Live Births in Brazil: Spatial and Professional Patterns, 2008–2023
Source: Public Health Chall. 2026 Oct 7;5(4):e70387. doi: 10.1002/puh2.70387 (PMC13645962; doi:10.1002/puh2.70387)
Supplement: Supplementary file 1 [file PUH2-5-e70387-s001.docx]

**Supplementary Table S1–** Absolute numbers of antenatal care consultations and live births by Brazilian state and study year, used to calculate the ANC/LB ratio presented in Table 1.

| Brazilian federative unit (state) | 2008 | | 2011 | | 2014 | | 2017 | | 2020 | | 2023 | |
| --- | --- | --- | --- | --- | --- | --- | --- | --- | --- | --- | --- | --- |
|  | ANC | LB | ANC | LB | ANC | LB | ANC | LB | ANC | LB | ANC | LB |
| RO | 74777 | 26791 | 152014 | 27658 | 168969 | 27560 | 101394 | 27503 | 33409 | 25798 | 35502 | 23921 |
| AC | 70039 | 17994 | 583583 | 17817 | 84823 | 17139 | 77039 | 16358 | 31416 | 15142 | 30712 | 14473 |
| AM | 330215 | 75030 | 330475 | 76202 | 744916 | 81145 | 305963 | 78066 | 16739 | 75635 | 7483 | 70453 |
| RR | 43640 | 10103 | 76501 | 9945 | 47220 | 11120 | 134115 | 11737 | 692 | 13760 | 1077 | 13105 |
| PA | 1283256 | 151262 | 929718 | 141974 | 1577605 | 143503 | 910128 | 138684 | 172440 | 132938 | 20366 | 126152 |
| AP | 77544 | 15105 | 81757 | 15114 | 116890 | 16271 | 48794 | 15399 | 285 | 14633 | 27 | 12948 |
| TO | 363900 | 25713 | 143740 | 25035 | 254340 | 24944 | 119024 | 24935 | 22404 | 23729 | 10226 | 23145 |
| MA | 1806711 | 128302 | 1498214 | 121109 | 1378856 | 117071 | 948163 | 112985 | 305588 | 106079 | 113767 | 97205 |
| PI | 165748 | 52664 | 346774 | 50144 | 385498 | 47941 | 271099 | 48551 | 4620 | 45229 | 2649 | 42144 |
| CE | 561081 | 133917 | 734379 | 128592 | 1143536 | 128681 | 1657493 | 127797 | 62162 | 121904 | 27481 | 111091 |
| RN | 243943 | 49817 | 609010 | 48101 | 216519 | 48111 | 245241 | 46222 | 141383 | 43531 | 6657 | 39440 |
| PB | 520941 | 61964 | 474434 | 58741 | 537816 | 57535 | 215349 | 57493 | 17304 | 56379 | 15613 | 51531 |
| PE | 686918 | 145195 | 1290093 | 140079 | 1438259 | 143489 | 649106 | 135932 | 64825 | 128481 | 47567 | 116175 |
| AL | 291953 | 58076 | 267380 | 54281 | 337707 | 51859 | 141537 | 50368 | 3432 | 48341 | 2187 | 46543 |
| SE | 626340 | 36633 | 141306 | 34925 | 318785 | 34369 | 148611 | 33867 | 35543 | 31784 | 14831 | 29005 |
| BA | 1692435 | 221700 | 2460650 | 215032 | 2249421 | 204034 | 1495147 | 204096 | 78001 | 188960 | 25062 | 170314 |
| MG | 1433649 | 260916 | 1481633 | 259863 | 3275253 | 267130 | 1795086 | 260959 | 583860 | 247198 | 312188 | 233918 |
| ES | 197362 | 51852 | 333584 | 53053 | 712580 | 56548 | 271106 | 55846 | 177595 | 53767 | 34986 | 52187 |
| RJ | 989983 | 215844 | 759754 | 220603 | 1061110 | 233584 | 1149516 | 223224 | 421676 | 199124 | 936874 | 176145 |
| SP | 3908511 | 601795 | 2955686 | 610222 | 4877135 | 625687 | 4004681 | 611803 | 1956784 | 552310 | 1589318 | 503910 |
| PR | 580688 | 151092 | 972114 | 152902 | 887766 | 159915 | 1514361 | 157701 | 452246 | 146291 | 492170 | 139836 |
| SC | 398110 | 85262 | 939601 | 87481 | 1153847 | 93232 | 475224 | 98335 | 257510 | 97916 | 291180 | 96802 |
| RS | 426622 | 135143 | 658506 | 137710 | 1777530 | 143315 | 471360 | 141568 | 203520 | 130742 | 157118 | 120974 |
| MS | 176900 | 41229 | 168687 | 42152 | 209854 | 44058 | 204183 | 44747 | 83474 | 41308 | 62223 | 40233 |
| MT | 561214 | 49794 | 578988 | 51218 | 1150109 | 56499 | 444179 | 57271 | 140017 | 57037 | 80689 | 58553 |
| GO | 325307 | 87462 | 453385 | 89742 | 1635232 | 99798 | 430277 | 97520 | 102627 | 92768 | 144955 | 91822 |
| DF | 300142 | 44173 | 196916 | 43465 | 203636 | 44721 | 236914 | 44568 | 23317 | 39361 | 15842 | 35551 |
